# Metaphern am Lebensende: Resilienz als Widerstand oder Akzeptanz

**DOI:** 10.1007/s00482-023-00702-z

**Published:** 2023-03-14

**Authors:** Veronika Koller

**Affiliations:** Department of Linguistics and English Language, Lancaster University, LA1 4YL Lancaster, Großbritannien

**Keywords:** Empowerment, Betreuung am Lebensende, Kommunikation Patient*innen, Kommunikation des medizinischen Personals, Kommunikation Angehöriger, Empowerment, End-of-life care, Patients/communication, Healthcare professionals/communication, Family members/communication

## Abstract

Der Beitrag bietet eine Übersicht über das Forschungsprojekt „Metaphern in der Betreuung am Lebensende“, das von 2011 bis 2014 an der Universität Lancaster in Großbritannien durchgeführt wurde. Zielsetzung des Projekts war es zu zeigen, a) wie Patient*innen, Familienangehörige und Gesundheitspersonal Metaphern verwenden, um über ihre Erfahrungen mit und Erwartungen an Betreuung am Lebensende zu sprechen, b) auf welche Erfahrungen und Bedürfnisse dieser Metapherngebrauch hindeutet und c) welchen Beitrag zur Kommunikation im Gesundheitswesen die Ergebnisse leisten können. Ein Korpus, bestehend aus Interviews mit verschiedenen Personengruppen und deren Beiträgen in Internetforen, wurde manuellen und halbautomatischen Analysen unterzogen. Die Ergebnisse zeigen, dass insbesondere die Onlinekommunikation von Patient*innen und Familienangehörigen von Gewalt- und Reisemetaphern geprägt ist. Der Metapherngebrauch dieser Gruppen deutet auf ein Bedürfnis nach Solidarität und Gemeinschaft hin und reflektiert sowohl positive als auch negative Selbsterfahrungen. Resilienz kann als Widerstand gegen Krankheit oder als deren Akzeptanz verstanden werden.

## Abstract (en)

The article provides an overview of the research project “Metaphor in end-of-life care”, which was conducted at Lancaster University (UK) between 2011 and 2014. The goals of the project were to show a) how patients, family members and healthcare professionals use metaphors to talk about their experiences with and expectations of end-of-life care, b) what experiences and needs are suggested by the use of metaphors by these groups and c) what contribution the results can make to communication in the healthcare system. A corpus consisting of interviews with the various groups of people and their postings in online fora were subject to both manual and semi-automated analyses. The results show that especially the online communications by patients and family members are characterized by violence and journey metaphors. The use of metaphors by these groups suggests a need for solidarity and community and reflects both positive and negative personal experiences. Resilience can be understood as resistance against illness or as acceptance of it.

Dieser Beitrag bietet einen Überblick über das Forschungsprojekt „Metaphern in der Betreuung am Lebensende“ („Metaphor in end-of-life care“), das von 2011 bis 2014 an der Universität Lancaster in Großbritannien durchgeführt wurde. Metaphern lassen sich definieren als das Sprechen, Schreiben und potenziell Denken über eine Sache mithilfe einer anderen Sache [12]. Oft werden Metaphern verwendet, um über subjektive, komplexe und sensible Erfahrungen, beispielsweise Gesundheit und Krankheit, zu sprechen. Dementsprechend gibt es eine umfangreiche Literatur zum Gebrauch von Metaphern in Diskursen zu Gesundheit und Krankheit, wobei sich vier Hauptstränge unterscheiden lassen [3]:

- Metaphern in der öffentlichen Kommunikation, etwa in Medienberichten
- Berichte über das individuelle Erleben von Krankheit
- Metaphern in der Kommunikation verschiedener Akteure, beispielsweise Ärzt*innen und Patient*innen
- Metaphern als Instrument in der Gesundheitskommunikation

Eine wichtige Erkenntnis dieser Forschung ist, dass Metaphern auch beeinflussen können, wie eine Krankheit erlebt wird [3]. Die beiden folgenden Beispiele aus den Projektdaten dienen als Illustration:

(1) „You have a lot to dig in and *fight* for and I know you can and will. Dust yourself down and prepare for the *battle* girl.“^1^

(2) „So sorry to hear what your partner is *going* through. MM [malignant melanoma] is a hard *road* to *travel* both physically and mentally.“

Die Beispiele stammen aus Internetforen für Patient*innen bzw. deren Angehörige. Im ersten Fall dienen die metaphorischen Ausdrücke – in beiden Beispielen kursiviert – dazu, jemandem Mut zuzusprechen, während im zweiten Beispiel Empathie vermittelt wird. Auch die Metaphern selbst unterscheiden sich voneinander: In Beispiel (1) realisieren die entsprechenden Wörter eine Gewaltmetapher, während Beispiel (2) eine Reisemetapher zugrunde liegt. Auf diese Weise werden sehr unterschiedliche Bedeutungen konstruiert, wie man die eigene Krankheit oder die von Angehörigen verstehen, über sie sprechen und sie erleben kann, nämlich entweder als Kampf gegen einen Gegner oder als Reise, auf der man sich befindet.

Vor allem häufig verwendete Metaphern sind fest in Diskursen verankert und ändern sich sehr langsam

Vor allem häufig verwendete und daher konventionell gewordene Metaphern sind fest in Diskursen verankert und ändern sich nur sehr langsam. Daher ist das hier vorgestellte Forschungsprojekt nach wie vor relevant, auch wenn es bereits 2014 abgeschlossen wurde und zum Teil auf früheren Daten basiert.

## Fragestellung

Das hier vorgestellte Forschungsprojekt beruht auf der Annahme, dass Metaphern einen Einblick in die Ansichten und Bedürfnisse von Sprecher*innen gewähren, im vorliegenden Fall von Patient*innen, Familienangehörigen und Gesundheitspersonal. Ebenso können potenzielle Ängste und/oder Missverständnisse durch Metaphern zutage treten. Wie im Folgenden gezeigt wird, können Metaphern daher auf verschiedene Arten Resilienz fördern oder erschweren. Resilienz wird hier verstanden als eine flexible Reaktion auf schwierige Umstände, bei der die Identität einer Person oder Gruppe bewahrt wird [15].

Basierend auf den obigen Annahmen stellt dieser Beitrag folgende Fragen:

1. Welche Metaphern verwenden Patient*innen, Familienangehörige und Gesundheitspersonal, um über ihre Erfahrungen mit und Erwartungen an die Betreuung am Lebensende zu sprechen? Wie werden diese Metaphern verwendet?
2. Auf welche Erfahrungen und Bedürfnisse deutet der Metapherngebrauch dieser Personengruppen hin?
3. Welchen Beitrag kann das Projekt zur Kommunikation im Gesundheitswesen leisten?

Im nächsten Abschnitt werden die Materialien und Methoden vorgestellt, die zur Beantwortung dieser Fragen herangezogen wurden.

## Material und Methoden

Für das Projekt „Metaphern in der Betreuung am Lebensende“ wurden Daten von drei Gruppen – Patient*innen, Familienangehörigen und Gesundheitspersonal – und aus zwei verschiedenen Textsorten verwendet: halbstrukturierten Interviews und Postings in Internetforen. Tab. 1 bietet eine Übersicht.

**Table 1:** 

	Patient*innen	Familienangehörige	Gesundheitspersonal	Insgesamt
Interviews	100.859	81.564	89.943	272.366
Internetforen	500.134	500.256	253.168	1.253.558
Insgesamt	600.993	581.820	343.111	*1.525.924 Wörter*

Für das Projekt wurden im Jahr 2012 Interviews mit 16 leitenden Mitarbeiter*innen in Hospizen und in der klinischen Palliativbetreuung durchgeführt. Im Gegensatz dazu stammen die Interviews mit 29 Patient*innen mit Krebs im Endstadium aus dem Projekt „Ethnicity and Cancer Care“ und die Interviews mit 17 pflegenden Familienangehörigen aus der Evaluierung des „Help the Hospices Major Grants Programme for Carers“. (Beide Projekte standen unter der Leitung von Sheila Payne und wurden im Zeitraum 2005–2009 bzw. 2006–2008 durchgeführt.) Bei der Datenerhebung in Internetforen ergab sich das Problem, dass es nicht genug öffentlich zugängliches Material für die Gruppe des Gesundheitspersonals gab; dieser Teil des Datensatzes wurde deswegen ergänzt durch Blogs von Ärzt*innen und Kommentare zu Beiträgen im *British Medical Journal*. Die Zahl der Individuen in den Internetforen lässt sich nicht mit Bestimmtheit feststellen, da es möglich ist, dass ein und dieselbe Person unter verschiedenen Namen postet. Davon abgesehen lassen sich Onlineforen als Erzählraum, jedoch nicht unbedingt als Dialograum verstehen; es ist daher nicht überraschend, eine Segregation verschiedener Gruppen in unterschiedlichen Foren zu finden.

Die Datenanalyse erfolgte in drei Schritten: Zunächst wurde eine Stichprobe von 92.000 Wörtern aus dem Gesamtkorpus einer manuellen Analyse unterzogen, unter Verwendung einer etablierten Methode zur Bestimmung von Metaphern [9]. Nach dieser Methode wird geprüft, ob die Bedeutung, die ein Wort im Kontext seiner Verwendung hat, von einer fundamentaleren, das heißt konkreteren oder älteren Bedeutung abweicht. Wenn dies der Fall ist und die kontextuelle Bedeutung sich durch einen Vergleich mit der fundamentalen Bedeutung erschließen lässt, dann handelt es sich um eine Metapher. In diesem ersten Schritt wurden alle metaphorischen Ausdrücke erfasst. Anschließend wurde eine halbautomatische semantische Annotation des gesamten Textkörpers vorgenommen. Dafür wurde das semantische Annotationsprogramm USAS (Lancaster University, Lancaster, UK) verwendet, das Teil der webbasierten Anwendung Wmatrix ist [10]. USAS beruht auf einem manuell erstellten Lexikon, das über Jahre von einer Gruppe von Forscher*innen ergänzt wurde. Diesem Lexikon zufolge wird jedem Wort in einer Textsammlung zumindest ein Bedeutungsfeld zugewiesen. Im zweiten Analyseschritt wurde festgestellt, zu welchem semantischen Feld die im ersten Schritt bestimmten metaphorischen Ausdrücke gehören und welche anderen Wörter sich im gleichen Feld befinden. Das Wort „battle“ (Schlacht) beispielsweise gehört zur Domäne „Kriegsführung“, die auch die Wörter „fighter“ (Kämpfer), „attack“ (angreifen, Angriff) und andere umfasst. Der letzte Schritt bestand in einer manuellen Analyse der Wörter aus verschiedenen Bedeutungsfeldern in ihrem sprachlichen Umfeld mit Hinblick auf etwaigen metaphorischen Gebrauch. Dieser Schritt ermöglichte es festzustellen, wie oft ein bestimmtes Wort im Gesamtkorpus metaphorisch gebraucht wird und welche anderen Wörter der gleichen Metapher zugeordnet werden können. Die sowohl manuelle als auch halbautomatische Methodik ermöglicht somit einen breiten Überblick über die Verwendung relevanter Metaphern in den Daten. Der nächste Abschnitt präsentiert die mit diesen Methoden gewonnenen Ergebnisse.

## Ergebnisse

### Quantitative Analyse

Der quantitative Teil der Analyse untersucht, welche Personengruppe welche Metaphern wie oft verwendet. Tab. 2 zeigt, dass vor allem Patient*innen und Angehörige Metaphern wesentlich häufiger in Internetforen als in Interviews gebrauchen; dies kann vielleicht damit erklärt werden, dass die schriftliche, asynchrone Kommunikation in Onlineforen zumindest potenziell einen bewussteren Sprachgebrauch erlaubt. Die relativen Häufigkeiten der Metaphern unterscheiden sich hingegen nur wenig voneinander, wenn man die Personengruppen vergleicht: 4,7 für Patient*innen und jeweils 4,2 für Familienangehörige und für das Gesundheitspersonal. Es ist jedoch bemerkenswert, dass Gewaltmetaphern (z. B. „fight“) allgemein den ersten Rang einnehmen, gefolgt von Reisemetaphern (z. B. „travel“). Dabei ist allerdings zu beachten, dass Letztere nicht ausschließlich wie im Abschnitt „Material und Methoden“ ausgeführt bestimmt wurden. Aufgrund der Tatsache, dass das Englische einige hochfrequente Wörter aufweist, die sich auf Bewegung beziehen, z. B. „through“ oder „going to“, wurden hier nicht alle Wörter in den entsprechenden Bedeutungsfeldern untersucht. Wenn es zeitlich und personell möglich gewesen wäre, Reisemetaphern vollkommen systematisch zu analysieren, hätten sie vermutlich den ersten Rang als am häufigsten verwendete Metaphern eingenommen.

**Table 2:** 

	Patient*innen			Familienangehörige			Gesundheitspersonal			Insgesamt
	Interview	Forum	Insgesamt	Interview	Forum	Insgesamt	Interview	Forum	Insgesamt	
Gewalt (siehe Abschnitt „Qualitative Analyse“)	72 (0,71)	899 (1,79)	971 (1,61)	80 (0,98)	807 (1,61)	887 (1,52)	73 (0,81)	337 (1,33)	410 (1,19)	**2268 (1,48)**
Reise (siehe Abschnitt „Qualitative Analyse“)	56 (0,55)	730 (1,45)	786 (1,3)	28 (0,34)	384 (0,76)	412 (0,7)	98 (1,08)	217 (0,85)	315 (0,92)	**1513 (0,99)**
Einschränkung („I feel so *trapped*“)	29 (0,29)	257 (0,51)	286 (0,47)	34 (0,41)	176 (0,35)	210 (0,36)	57 (0,63)	169 (0,67)	226 (0,66)	722 (0,47)
Tiere („the *beast* is back“)	18 (0,18)	120 (0,24)	138 (0,23)	9 (0,11)	397 (0,79)	406 (0,69)	2 (0,02)	48 (0,19)	50 (0,14)	594 (0,39)
Offenheit („things are out in the *open*“)	13 (0,13)	186 (0,37)	199 (0,33)	17 (0,2)	146 (0,29)	163 (0,28)	53 (0,59)	148 (0,58)	201 (0,58)	563 (0,37)
Sport und Spiel („a *marathon* rather than a *sprint*“)	1 (0,01)	124 (0,25)	125 (0,21)	5 (0,06)	72 (0,14)	77 (0,15)	18 (0,2)	50 (0,98)	68 (0,19)	270 (0,18)
Religion und Magie („try and kill my *dragons*“)	1 (0,01)	139 (0,28)	140 (0,23)	0	92 (0,18)	92 (0,16)	2 (0,02)	24 (0,09)	26 (0,07)	258 (0,17)
Hindernisse („a *barrier* to communication“)	5 (0,49)	71 (0,14)	76 (0,13)	7 (0,08)	56 (0,11)	63 (0,11)	24 (0,27)	62 (0,24)	86 (0,25)	225 (0,14)
Vollständigkeit („you are only *half* a person“)	4 (0,39)	58 (0,11)	62 (0,1)	3 (0,04)	62 (0,12)	65 (0,11)	16 (0,18)	16 (0,06)	32 (0,09)	159 (0,1)
Maschinen („back on the *treadmill* of treatment“)	10 (0,09)	29 (0,06)	39 (0,06)	4 (0,05)	36 (0,07)	46 (0,07)	23 (0,25)	12 (0,05)	25 (0,1)	114 (0,07)
Insgesamt	209 (2,1)	2613 (5,2)	**2822 (4,7)**	187 (2,3)	2228 (4,5)	**2415 (4,2)**	366 (4,1)	1083 (4,3)	**1449 (4,2)**	–
**Alle Metaphern**	–									**6686 (4,4)**

Dessen ungeachtet ist die Gewaltmetapher mit Abstand eine der am häufigsten verwendeten Metaphern im Gesamtkorpus. Gleichzeitig ist ihr Gebrauch aber auch umstritten [6, 14], weil sie das wohl größte Potenzial hat, das Verständnis und Erleben der letzten Lebensphase negativ zu beeinflussen. Diese Bedenken können auch erklären, warum das Gesundheitspersonal zumindest in dieser Untersuchung relativ gesehen signifikant weniger Gewaltmetaphern als Patient*innen verwendet (1,19 pro 1000 Wörter, im Gegensatz zu 1,61; *p* < 0,0001). Dieser vergleichsweise geringe Gebrauch, besonders in Interviews, weist auf den Einfluss von Grundsatzpapieren im britischen Gesundheitswesen hin [4], in denen Gewaltmetaphern bewusst vermieden werden. Auch ist es möglich, dass Ärzt*innen und Pfleger*innen weniger über die Themen sprechen, für die Patient*innen solche Metaphern verwenden, z. B. persönliche Resilienz. In diesem Zusammenhang lohnt sich ein Blick auf die verschiedenen Wörter, durch die die Gewaltmetapher ausgedrückt wird. Es zeigt sich, dass insbesondere das Wort „fight“ (Kampf, kämpfen) wesentlich häufiger von Patient*innen und Familienangehörigen verwendet wird (*p* < 0,05). Die folgende qualitative Analyse wird versuchen, diese Unterschiede zu erklären; der Schwerpunkt liegt dabei auf der internetbasierten Kommunikation zwischen Patient*innen.

### Qualitative Analyse

Der Gebrauch der im Textkörper so prominent vertretenen Gewaltmetapher ist vor allem deshalb umstritten, weil ein Verständnis von unheilbarer Krankheit als einen zu bekämpfenden Gegner dazu führen kann, dass Patient*innen die Schuld an einem „verlorenen“ Kampf zugeschrieben wird; siehe Beispiel (9). Im Folgenden wird sich zeigen, dass der Gebrauch von Metaphern im vorliegenden Korpus wesentlich komplexer ist; zunächst jedoch bedarf es einer Definition von Bestärkung und Entmachtung („empowerment“ und „disempowerment“). Diese können verstanden werden als Erweiterung bzw. Verengung des Handlungsspielraums, z. B. durch Ereignisse außerhalb der persönlichen Kontrolle. Alternativ können Bestärkung und Entmachtung auch als Kontrolle über Ereignisse bzw. als Mangel an Kontrolle aufgefasst werden. Entscheidend ist in beiden Fällen, dass der Handlungsspielraum oder die Kontrolle von der jeweiligen Person gewünscht und zu ihrem Wohl verwendet wird. Individuell gesehen kann Resilienz Widerstand gegen eine unheilbare Krankheit bedeuten, aber auch Akzeptanz von Krankheit und Lebensende. Eine Definition von Resilienz als „Widerstandsfähigkeit gegenüber krisenhaften Situationen und Lebensereignissen“ [5] ist daher irreführend: Obwohl Resilienz „auch mit einem kämpferischen Moment verbunden“ [15] sein kann, ist Akzeptanz, verstanden als ein aktives Annehmen unabänderlicher Umstände, ein wichtiger Faktor in der Resilienzbildung [11].

Die im Korpus dominierenden Gewalt- und Reisemetaphern werden von den verschiedenen Gruppen mit potenziell bestärkender Funktion gebraucht, können allerdings auch Ausdruck des Gefühls der Ohnmacht sein. Ein Blick auf die Daten zeigt, dass die Gewaltmetapher potenziell bestärkend ist, wenn Patient*innen erfolgreich gegen die Krankheit kämpfen. Die folgenden Beispiele illustrieren diese Funktion:

(3) „I don’t intend to give up; I don’t intend to give in. No, I want to *fight* it. I don’t want it to *beat* me, I want to *beat* it.“

(4) „Your words though have given me a bit more of my *fighting* spirit back. I am ready to *kick* some cancer butt!“

Beispiel (3) ist eine klare Willens- und Absichtserklärung, und insbesondere die grammatikalische Umkehrung im letzten Satz zeigt, dass die Absicht, ihre Krankheit zu bekämpfen („fight“) und zu besiegen („beat“), von der Patientin als positiv und bestärkend erlebt wird. Beispiel (4) erwähnt den in den Daten viel beschworenen Kampfgeist („fighting spirit“) und personifiziert die Krankheit. Hier wird verdeutlicht, dass Gewaltmetaphern zur gegenseitigen Ermutigung und Solidarität gebraucht werden können; siehe auch Beispiel (1). In einer bedenklich stimmenden Variante kämpfen sowohl Patient*innen als auch Familienangehörige erfolgreich gegen das medizinische Personal, z. B. um Informationen oder Termine zu erhalten. Dies weist auf Mängel im (britischen) Gesundheitssystem hin.

Auch die Reisemetapher kann eine bestärkende Funktion haben, vor allem wenn sie Szenarien hervorruft, in denen Patient*innen die Reise kontrollieren oder ihr positive Aspekte abgewinnen können, z. B. ein größeres Bewusstsein für ihr Leben:

(5) „My *journey* may not be smooth but it certainly makes me look up and take notice of the *scenery*!“

Gemeinschaft und Solidarität spielen eine wichtige Rolle, wenn Patient*innen einander als Reisegefährt*innen verstehen, die einander helfen, Schwierigkeiten zu überwinden:

(6) „*Rocks* in our *paths* are easier to handle when we’re all in it together.“

Andererseits können sowohl die Reise- als auch die Gewaltmetapher Ausdruck von Ohnmacht sein. Erstere kann ein Szenario entwerfen, in dem Patient*innen keine Kontrolle über die Reise haben oder die Reise so beschwerlich ist, dass keine positiven Aspekte erkennbar sind.

(7) „How the hell am I supposed to know how to *navigate* this *road* I do not even want to be on?“

(8) „[Having cancer] is like trying to *drive* a *coach* and *horses uphill* with no *back wheels* on the *coach*.“

Auch der Gebrauch von Gewaltmetaphern kann Patient*innen als machtlos konstruieren. Dies kann in verschiedenen Szenarien geschehen: Zunächst kann der Kampf gegen die Krankheit nicht erfolgreich sein, wofür Patient*innen sich selbst die Schuld geben:

(9) „I feel such a failure that I am not winning this *battle*.“

Ferner können Patient*innen sich als machtlos angesichts eines gewaltsamen Gegners erleben. Insbesondere im Fall von Krebs werden zum Teil auch die Nebenwirkungen von Chemo- und Strahlentherapie als Feind verstanden:

(10) „But the emotional side of cancer … is the real *killer*—it *strangles* and shocks your soul.“

(11) „What did I think all my normal little cells were doing after being *hit* by a *sledgehammer* of both toxic chemicals and radiation?“

Und letztlich kann auch der als Kampf verstandene Umgang mit dem medizinischen Personal als Machtlosigkeit erlebt werden, z. B. wenn bestimmte Medikamente – in Beispiel (12) Rüstung („armour“) genannt – aus Kostengründen nicht verfügbar sind:

(12) „It must be dispiriting when you are *battling* as hard as you can, not to be given the *armour* to *fight* in.“

Angesichts dieser Ergebnisse stellt sich die Frage, welche der beide Metaphern insbesondere für Patient*innen ein eher bestärkendes oder eher entmachtendes Potenzial hat. Eine Zufallsstichprobe von 100 Gewaltmetaphern im entsprechenden Teilkorpus zeigt, dass 42 in potenziell bestärkender Art verwendet werden, wohingegen 38 die Patient*innen als ohnmächtig darstellen (20 können keiner der beiden Kategorien zugeordnet werden). Im Gegensatz dazu wird die Reisemetapher in 39 Fällen als Ausdruck der Ohnmacht, aber nur in 26 Fällen in potenziell bestärkender Funktion verwendet (35 Beispiele zeigen keine eindeutige Funktion). Es lässt sich also festhalten, dass – im Gegensatz zur Überzeugung vieler Ärzt*innen und Pfleger*innen – Patient*innen und auch Angehörige die Gewaltmetapher regelmäßig in einer potenziell bestärkenden Art verwenden und daher möglicherweise auch so verstehen und erleben. Dabei bedienen sie sich eines breiten Spektrums an Ausdrücken („aggressive“, „fight“, „kick“ und „war“), um über eine Bandbreite von Erfahrungen mit Betreuung am Lebensende zu sprechen. Gewaltmetaphern dienen insbesondere dem Ausdruck persönlicher Entschlossenheit, Solidarität und Ermutigung, und obwohl die Szenarien variieren, bleibt festzuhalten, dass nicht alle Gewaltmetaphern eine negative Wirkung haben müssen.

Die identitätsstiftende oder -stärkende Funktion von Metaphern ist in der Literatur umfassend diskutiert worden, auch in Bezug auf Krankheit und Schmerz [2, 7]. Im Fall der Gewaltmetapher wird diese im vorliegenden Textkörper u. a. durch das Wort „fighter(s)“ (Kämpfer) realisiert. Dies geschieht ausschließlich mit positiver Bewertung, sei es zur Selbstbeschreibung, zur Beschreibung anderer oder zur Beschreibung einer Gruppe:

(13) „My consultants recognized that I was a born *fighter* and saw my determination to prove them wrong.“

(14) „You are such a *fighter* and so inspirational.“

(15) „I’m new to the forum and wanted to know if there are any other younger bowel cancer *fighters* amongst us.“

Auch die Reisemetapher kann als Identitätsmerkmal gebraucht werden, z. B. wenn andere Mitglieder in einem Internetforum als „fellow travellers“ (Mitreisende) begrüßt werden. Die Identität als Reisende*r kann wiederum bestärkend sein:

(16) „We all need to choose our own *road* through this … , not to be a passive patient but to feel an empowered collaborator in our *road* *back* to full health.“

Andererseits jedoch kann die Reisemetapher, gerade im Diskurs zum Lebensende, ein Szenario entwerfen, in dem der Tod den Endpunkt der Reise darstellt:

(17) „It saddens my heart to read of the *passengers nearing* the *end* of their *journey* and those recently having finished their *journey*.“

Zusammenfassend lässt sich sagen, dass Metaphern in der Onlinekommunikation unter Patient*innen verwendet werden, um verschiedene individuelle und Gruppenidentitäten auszudrücken, inklusive Veränderungen in Identität und (Selbst‑)Wahrnehmung während des Krankheitsverlaufs. Metaphern dienen weiter dazu, Rat, Unterstützung und Empathie zu kommunizieren sowie Beziehungen und Gemeinschaften zu bilden und zu erhalten. Die qualitative Analyse liefert somit weitere Belege für die Rolle von Internetforen als Ort, an dem Patient*innen Gefühle zulassen können und Unterstützung, Rat, Empathie und Solidarität finden.

## Diskussion

In Beantwortung der im Abschnitt „Fragestellung“ formulierten Forschungsfragen lässt sich Folgendes konstatieren: Patient*innen, Familienangehörige und Gesundheitspersonal verwenden eine Reihe von Metaphern, um über ihre Erfahrungen mit und Erwartungen an die Betreuung am Lebensende zu sprechen. Gewalt- und Reisemetaphern sind am häufigsten anzutreffen, vor allem im Sprachgebrauch von Patient*innen und Familienangehörigen und hier besonders in Internetforen. Beide Metaphern können potenziell bestärkend wirken, aber auch Ausdruck von Ohnmacht sein, wobei sich die jeweiligen Funktionen teilweise an bestimmten Wörtern (z. B. „fighter“) festmachen lassen.

Gewalt- und Reisemetaphern können potenziell bestärkend wirken, aber auch Ausdruck von Ohnmacht sein

Des Weiteren dienen sowohl Reise- als auch Gewaltmetaphern der individuellen und kollektiven Identitätsbildung. Der Metapherngebrauch von Patient*innen und Angehörigen deutet auf ein Bedürfnis nach Solidarität und Gemeinschaft hin und reflektiert sowohl positive als auch negative Selbsterfahrungen, sei es im Widerstand gegen die Krankheit oder in deren Akzeptanz. Der Gebrauch von Metaphern ist dabei beeinflusst von den miteinander verwobenen Erfahrungen, Vorlieben und Gewohnheiten der jeweiligen Individuen und Gruppen. Wenn Resilienz entweder als Widerstand oder als Ergebung [1] verstanden werden kann, ist der hier untersuchte Diskurs – besonders der von Patient*innen – eher geprägt durch einen potenziell bestärkenden Gebrauch von Gewaltmetaphern, die das kranke Selbst als von der Gruppe unterstützte Kämpfer*in konstruieren.

Der Beitrag, den das Projekt zur Kommunikation im Gesundheitswesen leisten kann, wird im „Fazit für die Praxis“ thematisiert.

## Fazit für die Praxis

Das Projekt „Metapher in der Betreuung am Lebensende“ ist in einer Buchpublikation dokumentiert und hat ein ähnliches Projekt in Schweden inspiriert (Metaphern in der palliativen Krebsbetreuung, 2013–2016). Am wichtigsten für die Patientenbetreuung jedoch ist das sogenannte Metaphernmenü, eine illustrierte Sammlung von Zitaten von Krebspatient*innen, gedacht als Hilfe nach der Diagnose. Die Broschüre enthält einige Beispiele von Gewalt- und Reisemetaphern, aber auch Metaphern aus anderen Erfahrungsdomänen. Sie soll Gespräche zwischen Ärzt*innen und Patient*innen erleichtern. Auch bietet die Broschüre Letzteren verschiedene Möglichkeiten an, ihre Erkrankung zu verstehen und zu erleben. Für Ärzt*innen ist es in diesem Zusammenhang wichtig, ein Bewusstsein für den eigenen Sprachgebrauch zu entwickeln, sensibel auf die Erfahrungen, Gefühle und Bedürfnisse zu reagieren, die Patient*innen durch die Verwendung von Metaphern kommunizieren, und offen zu sein für Unterschiede im individuellen Sprachgebrauch.

## Footnotes

1 Im weiteren Sinn kann auch „dust yourself down“ als Teil der Gewaltmetapher verstanden werden. Es ist hier jedoch nicht kursiviert, weil diese Bedeutung nur implizit im Kontext vorhanden ist.
